# Supplementary material for: Altered microRNA Transcriptome in Cultured Human Liver Cells upon Infection with Ebola Virus
Source: Int J Mol Sci. 2021 Apr 6;22(7):3792. doi: 10.3390/ijms22073792 (PMC8038836; doi:10.3390/ijms22073792)
Supplement: Supplementary file 1 [file ijms-22-03792-s001.zip › Supplementary_File/C_ GO_Analysis_Results/16-30nt_go_Reston-24h-Huh7_vs_Control-24h-Huh7_up.mature_mirna_targets/MF_Count.pdf]

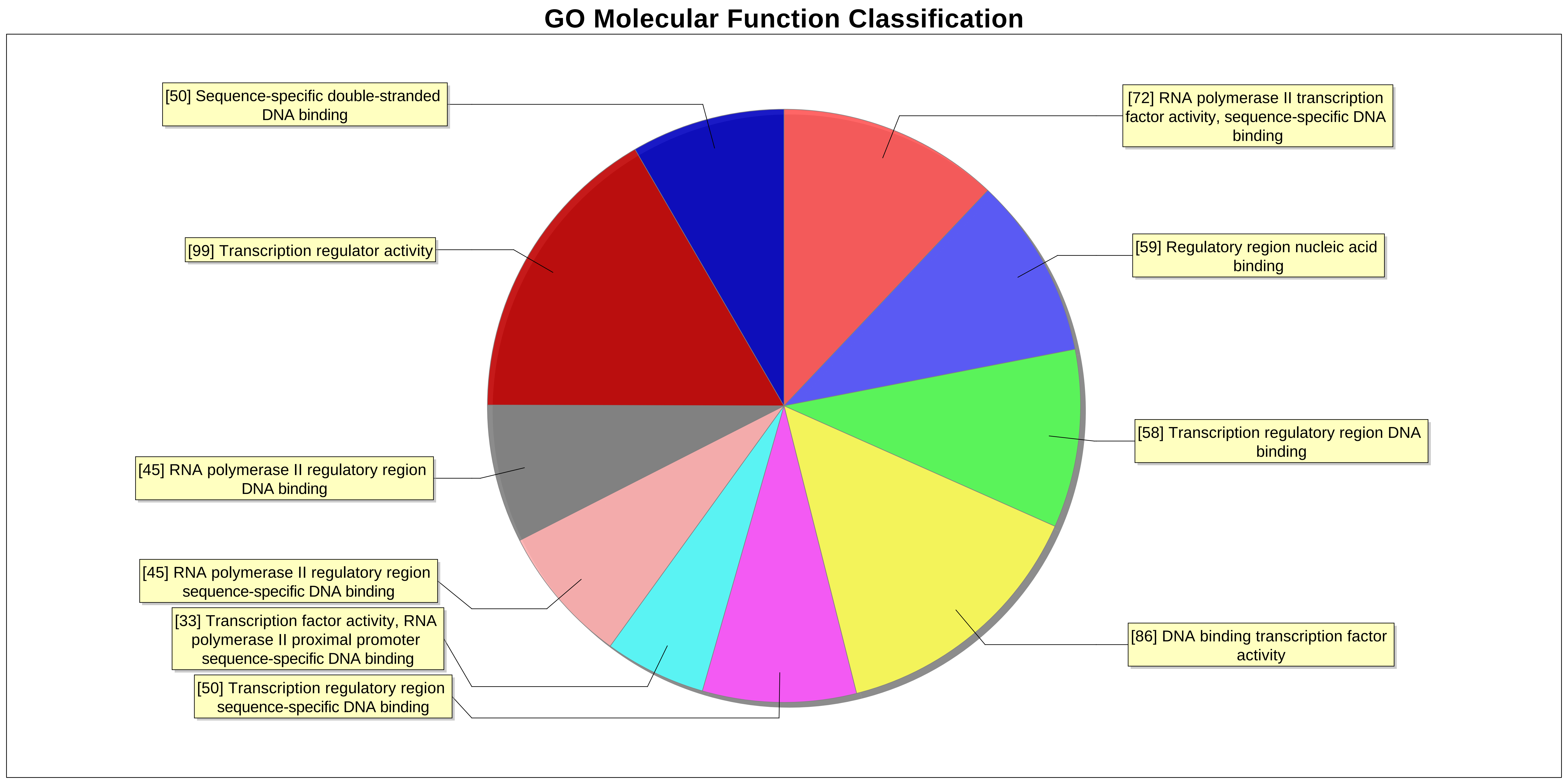
GO Molecular Function Classification
[50] Sequence-specific double-stranded DNA binding
[72] RNA polymerase II transcription factor activity, sequence-specific DNA binding
[99] Transcription regulator activity
[59] Regulatory region nucleic acid binding
[58] Transcription regulatory region DNA binding
[45] RNA polymerase II regulatory region DNA binding
[45] RNA polymerase II regulatory region sequence-specific DNA binding
[33] Transcription factor activity, RNA polymerase II proximal promoter sequence-specific DNA binding
[50] Transcription regulatory region sequence-specific DNA binding
[86] DNA binding transcription factor activity
